# Ash and Orihel’s Atlas for the Ages

**DOI:** 10.4269/ajtmh.20-0034

**Published:** 2020-02-10

**Authors:** Claire Panosian Dunavan

**Affiliations:** 1 University of California, Los Angeles, California, E-mail: cpanosian@mednet.ucla.edu

“In this era of increased parasitic threat, **Human Parasitic Diseases - A Diagnostic Atlas** is a must-have for parasite identification. Coverage is complete, including well-recognized species of parasites as well as information on those less commonly encountered. This complete expansion of the classic Atlas of Human Parasitology is the perfect reference to have at hand when you need to view the unknown and assimilate your findings into a clinical context.”

—Editorial Review, Amazon, October 2019

“Working with parasites and finding out about them is so much fun that I still find it fun today [Larry Ash laughs].”

—Interview, Pasadena, CA, March 2019

In the living room of a one-story Monterey Colonial framed by a spreading oak, a cheerful 86-year-old sits perched on a director’s chair; behind him is a cinematographer’s background cloth, also known as a “green-screen.” UCLA’s beloved maven of all things protozoan and helminthic is ready for his interview about rat lungworm, an exotic beast whose life cycle he helped to decrypt. Over the next hour, he discusses his early fieldwork on *Angiostrongylus cantonensis*, both in Hawaii and French Polynesia, along with a lifelong fascination with parasites. Weaving through the narrative is a third, historical strand. How the heck did a biology major from Northampton, MA, who—until graduate school—had never ventured more than 200 miles from home (traveling to Michigan was his first “long-distance” adventure), come to embrace the vast kin of parasites—then, over decades, educate countless students and colleagues around the globe through lectures, atlases, and texts?

Later in this piece, certain serendipities propelling the career of Dr. Lawrence Ash, a long-time professor at the UCLA School of Public Health, will unfold. But first things first.

Several months after his initial visit, Larry Ash returned to our house with a hefty volume fresh from the printer. On its cover were geometric shapes of turquoise and blue and five of the 1,500 (count them!) high-quality images contained within. In short, the 2020 edition of Ash and Orihel’s *Human Parasitic Diseases—A Diagnostic Atlas* published by the American Society of Clinical Pathology—the culmination of a lifetime of collecting—is a 672-page feast for anyone who wishes to know parasites not merely as “research subjects” with receptors, molecules, and genes but as some of the most original and versatile members of the animal kingdom.

In addition to its vast cache of pictures, this latest offering from Ash and his long-time coauthor Dr. Thomas Orihel (1929–2019) features a well-designed layout; updated, user-friendly text; pictures of parasites in feces, blood, and lab preps; many photomicrographs of parasites in tissue (a great boon to clinical pathologists); illustrated keys; discussions of new serologic and molecular methods of diagnosing parasites; images of arthropods that transmit parasites; images of arthropod larvae that invade tissue; and even photos of artifactual items commonly mistaken for parasites. Accordingly, it’s no exaggeration to describe this book as an all-encompassing reference as well as a treasured possession for anyone in tropical medicine.

In its opening dedication, *Human Parasitic Diseases—A Diagnostic Atlas* also honors one of our own. Which brings us back to 1956 when Ash first visited the University of Michigan’s Biological Station. It was there he met Paul Beaver (1905–1993), Tulane’s then-incoming chairman of tropical medicine who later served a 12-year term as editor-in-chief of the *American Journal of Tropical Medicine and Hygiene*. If there is a pantheon for parasitologists, Beaver is surely among those elite. Although reserved by nature, he was famously supportive of students and knew talent when he saw it. Soon after meeting Ash, he quickly convinced young Larry to pull up stakes and move to Tulane where Beaver had recently succeeded the world-famous Ernest Carroll Faust.^1^ And thus, the adventures began.

“Paul Beaver was not only my mentor,” Ash says to this day. “He was my second father.”

Talk about a role model.

## A CONVERSATION WITH LARRY ASH, PhD^2^

**When did you first become interested in parasites?** As a biology major at the University of Massachusetts, I had not found anything that really turned me on until the second semester of my senior year. That’s when I took a course in parasitology from a lady who was elderly and somewhat eccentric. But she made parasites so interesting. Then, after finishing my bachelor’s degree, Dr. Jay Traver said: “Larry, what are your plans?” The truth is: I had no plans. “So why don’t you do a master’s with me?” she asked. “You can do a survey of parasites in Massachusetts.” I wound up trapping rodents.

What kept me fascinated was the broad array of parasites I found in rodents’ intestines, blood, and other organs. And because there was no Internet to consult back then, I learned about nematodes, cestodes, trematodes, and blood parasites by dissecting animals, finding their parasites, staining them, and then identifying their genus and species by tracking them down in the literature.

What also turned me on were parasites’ fascinating life cycles, each so different. *Angiostrongylus* is a classic example of something that uses snails as an intermediate host. Schisto also uses snails. Other things use snails in addition to other intermediate hosts, and you can’t help but wonder: how did this happen? In the case of Angio, how did rats come into the picture? Where did Angio come from in the first place?

**After living in New England your entire life, how did you like New Orleans?** I absolutely loved it. I also had great roommates from overseas: a Chinese roommate from Malaysia and another from Buenos Aires. From them, I learned that parasitologists are everywhere. There may not be many of us, but we are everywhere.

**Do you remember your first ASTMH conference?** It was either in 1956 or 1957 because it was held in New Orleans and we were beginning students attending our first-ever meeting. At Tulane, Paul Beaver and Ernest Carroll Faust had pictures of famous parasitologists dating back to the late 1800s and early 1900s. It was just thrilling to meet some of these people.

**Let’s switch gears to *Angiostrongylus*. What are your earliest memories and experiences with this parasite in particular?** I first heard about *Angiostrongylus* when I was in New Orleans, working on my PhD, and two parasitologists in Australia described it in rats in Brisbane. But what interested me most was the parasite’s migration through the rat’s brain. The two women scientists [Jo Mackerras and Dorothea Sandars] presented their findings in England and were ridiculed! But, of course, they turned out to be right. They were the first to discover that Angio went through the brain of rodents.

My own experience with Angio began after I completed my PhD and was working as a postdoc with Joseph Alicata [an established parasitologist at the University of Hawaii] surveying different mammals in the Hawaiian Islands. It was while I was looking for parasites in rodents, deer, dogs, cats, etc. that we first heard about cases of meningitis in people in Tahiti. At the time, no one knew the cause; local experts had looked and could not find parasites. It was only after a pathologist named Grant Stemmermann recalled two patients on Oahu who had died in a mental asylum in the 1950s that he went back, examined their brains, and found worms.

**What happened next?** Because Joseph Alicata and I were both interested in identifying parasites in human tissues, we examined the parasites Stemmermann had found. However, we did not know exactly what we were seeing until the brains were sent to Bethesda and examined by May Belle Chitwood. She identified them as larvae of *A. cantonensis.*

Once this was learned, the question arose: how did these people become infected? Because we already knew that the life cycle involved slugs and snails, I started [my research] in Honolulu by making slug milkshakes. You put the slugs into artificial gastric juice and dump them in a blender, then examine the sediment to find *Angiostrongylus* larvae.

In Angio, the adult worms live in the lungs and pulmonary arteries of rodents, rats especially. They produce eggs which become larvae in the lungs, and those larvae are excreted in rodent feces. The fecal material is ingested by slugs and snails. The larvae develop to the infective stage in about 3 weeks, and then rodents eating the slugs and snails become infected.

I was then sent to New Caledonia to figure out the epidemiology of human Angio infection and to work at the South Pacific Commission, which was a small United Nations established by the United States, Britain, New Zealand, Australia, and France. I was recruited by Leon Rosen, the head of the NIH’s Pacific Research Station in Hawaii, who had already started to investigate cases of eosinophilic meningitis in Tahiti. During my night runs, I would go out wearing a headlamp and collect snails and slugs in vegetable gardens because it was on lettuce and other green vegetation that the parasites and their intermediate hosts were present.

**Did you ever worry that you yourself might contract *Angiostrongylus*?** Personally, I’ve never acquired a parasite in all my years of working with them. But I do have a funny story. Leon Rosen came to New Caledonia about twice a year to see how I was doing. One night my wife Luana prepared dinner for him at our small apartment and gave him salad where he found a snail. My wife never got over being embarrassed by that [Ash laughs].

**Tell me more about the movement of *Angiostrongylus*. In your opinion, is it likely to continue spreading to new regions, especially coastal cities?** The geographic distribution of *A. cantonensis* is very interesting. The parasite probably originated in southeast China. During World War 2, when the Japanese were invading other Pacific islands, they brought snails with them as a food source. The Japanese introduced snails into many Pacific islands they conquered; that’s, in part, how *Angiostrongylus* spread throughout the Pacific.

At the same time, rats and mollusks also travel by ship and that accounts for the introduction of Angio into other geographic areas. For instance, throughout my years at Tulane, we never found *A. cantonensis*. But it was eventually introduced through the port of New Orleans and later moved into other parts of Louisiana. Now it’s present throughout much of the southeastern United States. Most cases seen in the continental United States are in kids who will put almost anything in their mouths; for them, slugs and snails are fair game. On the other hand, during the early outbreaks in Tahiti, it was freshwater shrimp containing larvae that infected people. In other Pacific islands, crabs were infected. We’ve even demonstrated *A. cantonensis* in the muscle of cows, so clearly different food products in different parts of the world can lead to transmission.

**What else would you like to say about larval burden in snails and clinical illness in humans?** In Tahiti, the original location, many individuals developed mild symptoms that would quickly disappear, or they would experience multiple infections but never suffer severe illness. But in other areas, it does appear that angiostrongyliasis is becoming more clinically severe. Is it a different strain of the parasite? We really do not know.

The large *Achatina* [African land snail] that you see in Hawaii and many other parts of the world can carry literally thousands of larvae. When you digest these large snails, it’s amazing how many larvae they contain. But smaller snails and slugs also serve as intermediate hosts of Angio and probably account for even more accidental ingestion by humans.

Finally, we still don’t know how often worms in human brains continue on to the lungs and pulmonary arteries. In the early days, we never saw that, but in recent years, we’ve seen more cases of larvae completing the migration to the lung and pulmonary arteries of people.

**What are some leading diagnostic challenges?** Diagnosis of Angio infection is a real problem because you never really know when you’ve ingested the larvae. And the early symptomatology—headaches, flu-like feelings, that sort of thing—can be seen in many other infections. It’s hard to diagnose angiostrongyliasis until the worms are already in the brain and causing serious problems.

[In addition,] many clinicians aren’t aware of *Angiostrongylus* in their areas, so they don’t think about it. By the time they do, it’s probably too late to treat. I really don’t know what to suggest other than clinicians becoming more aware of certain exotic infections in their own backyards.

**Let’s move on to your atlas. How did it start?** I became interested in teaching about techniques of diagnosis back in New Caledonia because the labs there didn’t know the best ways to find parasites. That led to a phone call in the mid-1970s from Dr. James Smith, a pathologist in Indiana, who wanted to develop a Kodachrome atlas to teach about parasites. And so, over time, six of us created three Kodachrome atlases. Eventually, Tom Orihel, and I thought: “Gee, if we’re making Kodachrome atlases maybe we should also develop a bench atlas that people can consult while they’re looking under the microscope.” And that led to the first *Atlas of Human Parasitology* in 1980, which was mainly for laboratory personnel trying to identify specimens. Ten or 15 years later, we developed a second book meant to help pathologists identify parasites that enter human tissues. Today, we have been through five editions of the first atlas along with this newest edition that shows parasites in tissue sections as well as what you might find in blood or fecal smears.

**I know that collecting specimens is another one of your passions. In fact, right now as we’re speaking in your UCLA office, I’m staring at a 12-ounce bottle labeled *Strongyloides* and *Opistorchis*, Thailand camp, April 14, 1977.** Well, I’ve been at UCLA now for some 52 years, and I have always been a collector or a hoarder of parasites—both animal and human. I always had many vials and jars of specimens so that people who came to visit my lab could see whole worms in bottles and that sort of thing. I had quite a collection.

For a long time, Tom [Orihel] and I also supplied ASCP and the American Society of Microbiology with parasite samples they sent out as unknowns. We made literally thousands of malaria slides—I still have some of them.

Just a few weeks ago, someone was out from CDC to visit my samples.^3^ Would you believe, it was the first time they were able to take photos of *Metagonimus*?

**What are some of your concerns for the future of parasitology?** What has happened in the teaching of parasitology today as opposed to, say, 40 or 50 years ago, is that medical school curricula have changed extensively. At the same time, there’s a general feeling that parasites are not a very big problem in this country, which I would question. But the teaching of parasitology is so far down that today, many physicians have never taken a class. How do you develop a differential diagnosis if you’ve never heard of a parasite that might be involved [in someone’s illness]? It’s a big problem.

Of course, another part of the problem relates to funding. Even if people are interested in parasitology, if they’re in an academic setting and they want to do research, they’re going to have to get money from the NIH or wherever. And while there’s tremendous interest in developing molecular tests to diagnose parasites, the ability to actually recognize parasites is fading away. In the future, there will be very few people who can recognize and identify parasites of animal or human origin or even teach parasitology. That’s a scary prospect.

**Do you have any solutions?** It’s hard to know… maybe foreign schools and labs? Here, you’ve got one or two hours to teach medical students about parasites. What do you say? I have no idea. Go read my book.

**In closing, please share a final anecdote about *Angiostrongylus*. I know you have many.** After I had finished my work in Hawaii and the laboratory shut down, I wanted to continue research on *Angiostrongylus* back in New Orleans, but it would have been awkward to carry the mollusks by hand. My lovely wife offered to carry several infected mollusks in a plastic bag in her bra. And that’s how *Angiostrongylus* got established in the laboratory at Tulane.

***Addendum:*** *Tom Orihel and Larry Ash were Paul Beaver’s first PhD students, and the two of them collaborated for over 63 years. Orihel died in December 2019 at age 90 years, but not before he was able to see the 2020 edition of Human Parasitic Diseases—A Diagnostic Atlas*.

## Footnotes

1 In 1928, when Ernest Carroll Faust, Ph.D. joined Tulane’s medical faculty as chair of parasitology, he had already spent nine years living and studying parasites in China. In 1929, he published *Human Helminthology*, the first English-language parasitology text. In 1937, his coauthored work with Charles Craig became another landmark book in the field.

2 This interview, which has been edited for length and clarity, contains portions of conversations recorded in March, November, and December 2019.

3 Larry Ash’s entire collection of parasite specimens will soon move to the Centers for Disease Control.

